# Cost-effectiveness analysis of pre-exposure prophylaxis for the prevention of HIV in men who have sex with men in South Korea: a mathematical modelling study

**DOI:** 10.1038/s41598-020-71565-y

**Published:** 2020-09-03

**Authors:** Heun Choi, Jiyeon Suh, Woonji Lee, Jun Hyoung Kim, Jung Ho Kim, Hye Seong, Jin Young Ahn, Su Jin Jeong, Nam Su Ku, Yoon Soo Park, Joon Sup Yeom, Changsoo Kim, Hee-Dae Kwon, Davey M. Smith, Jeehyun Lee, Jun Yong Choi

**Affiliations:** 1 Department of Internal Medicine and AIDS Research Institute, Yonsei University College of Medicine, Seoul, Republic of Korea; 2 Department of Computational Science and Engineering, Yonsei University, Seoul, Republic of Korea; 3 Department of Preventive Medicine, Yonsei University College of Medicine, Seoul, Republic of Korea; 4 Department of Mathematics, Inha University, Incheon, Republic of Korea; 5 Department of Medicine, University of California San Diego, La Jolla, CA USA; 6 Veterans Affairs San Diego Healthcare System, San Diego, CA USA; 7 Department of Mathematics, Yonsei University, Seoul, Republic of Korea; 8 Division of Infectious Diseases, Department of Internal Medicine, Yonsei University College of Medicine, Yonsei University Health System, 50-1, Yonsei-ro, Seodaemun-gu, Seoul, 03722 South Korea

**Keywords:** HIV infections, Preventive medicine

## Abstract

In February 2018, the Ministry of Food and Drug Safety in Korea approved tenofovir disoproxil fumarate and emtricitabine (TDF/FTC) co-formulate for use in pre-exposure prophylaxis (PrEP) for the prevention of human immunodeficiency virus (HIV) infection. This study aimed to estimate the cost-effectiveness of PrEP in men who have sex with men (MSM), a major risk group emerging in Korea. A dynamic compartmental model was developed for HIV transmission and progression in MSM aged 15–64 years. With a combined model including economic analysis, we estimated averted HIV infections, changes in HIV prevalence, discounted costs, quality-adjusted life-years (QALYs), and incremental cost-effectiveness ratios (ICERs). PrEP was evaluated in both the general MSM and high-risk MSM populations and was assumed to reduce infection risk by 80%. Implementing PrEP in all MSM would avert 75.2% HIV infections and facilitate a gain of 37,372 QALYs at a cost of $274,822 per QALY gained over 20 years relative to the status quo. Initiating PrEP in high-risk MSM with an average of eight partners per year (around 20% of MSM) would improve the cost-effectiveness, averting 78.0% HIV infections and add 29,242 QALYs at a cost of $51,597 per QALY gained, which is within the willingness-to-pay threshold for Korea of $56,000/QALY gained. This result was highly sensitive to annual PrEP costs, quality-of-life for people who are on PrEP, and initial HIV prevalence. Initiating PrEP in a larger proportion of MSM in Korea would prevent more HIV infections, but at an increasing cost per QALY gained. Focusing PrEP on higher risk MSM and any reduction in PrEP cost would improve cost-effectiveness.

## Introduction

According to UNAIDS, the number of new HIV infections in the world was down 35% in 2018 from 2000; however, the number of new annual infections in Korea increased from 244 to 1,206 over the same period, mainly among men who have sex with men (MSM). Pre-exposure prophylaxis (PrEP) with tenofovir disoproxil fumarate (TDF) and emtricitabine (FTC) may help reduce the HIV epidemic in Korea.

In the iPrEx study, 2,499 men and male-to-female transgenders who have sex with men were randomized to either a daily fixed-dose combination of TDF and FTC or a placebo. TDF/FTC group was associated with a 44% relative risk reduction in HIV acquisition, and the efficacy was estimated at > 90% among those with detectable blood drug levels^1,2^. With proven efficacy in HIV prevention, PrEP has been found to be safe and efficacious in various clinical trials and approved by the United States Food and Drug Administration (FDA) for use as PrEP in 2012^3–5^.

Although PrEP has been shown to be an effective preventive measure for HIV infection, its costs are considerable. A mathematical modeling study showed that use of PrEP for HIV prevention in the general population of MSM in the US could prevent a substantial number of HIV infections, and although PrEP use in high-risk MSM was considered most cost-effective, it was estimate to result in annual expenditures over $4 billion^6^. Further, a mathematical modeling study suggested that a combination of PrEP and early diagnosis of HIV infection could be a very effective way to reduce HIV incidence in South Korea among MSM^7^. Although TDF/FTC has been approved for use in PrEP by the Korean Ministry of Food and Drug Safety, the use of PrEP is not yet covered by Korean national health insurance, and the high cost of PrEP, at $480 per month for TDF/FTC can be a significant barrier for PrEP implementation in South Korea. In this study, a mathematical model of the HIV epidemic among MSM in South Korea was constructed to evaluate the cost-effectiveness and preventive effects of PrEP in terms of the MSM population in South Korea and drug costs.

## Methods

### Study design and model structure

**Figure 1:**
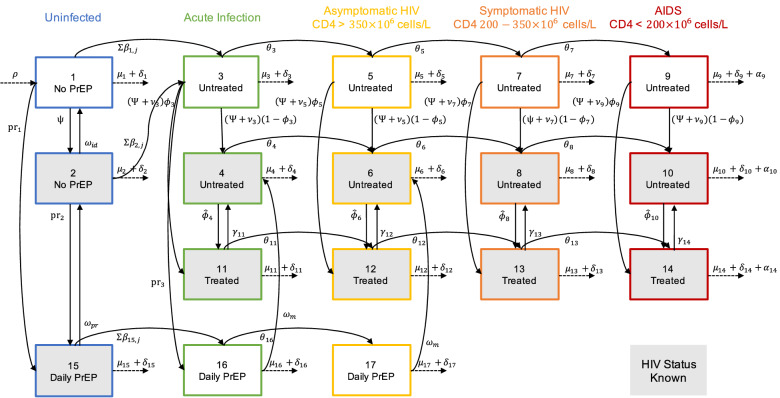
Multistate HIV infection model for MSM population. See Table S1 for the meaning of each abbreviation.

We constructed a dynamic compartmental model of HIV infections among MSM in South Korea to assess the cost-effectiveness of PrEP for HIV prevention within this population (Fig. 1). The model estimated HIV prevalence, incidence, quality-adjusted life-years (QALYs), and healthcare cost of PrEP strategies among MSM in South Korea over 20 years. Our base-case analysis estimates HIV infections averted and incremental cost-effectiveness ratios (ICERs) associated with implementing each PrEP strategy. All costs are presented in 2017 US dollars, which were 3% discounted annually. We programmed the model using MATLAB R2019a (MathWorks, Natick, Massachusetts).

We divided the population by HIV infection status, awareness of HIV status, and PrEP use. Persons with HIV were defined by HIV disease stage and treatment status. Initial HIV prevalence and annual incidence were considered as 7.95% and 1.38%, respectively^8–11^. We assumed a PrEP duration of 20 years, which is the same as the simulation time, and estimated the cost-effectiveness of PrEP in two groups: general MSM and high-risk MSM. The model was solved for each group in a different simulation by changing the values of following three parameters: total population sizes of 217,280 for general MSM and 43,456 for high-risk MSM; initial prevalences of 7.95% for general MSM and 15% for high-risk MSM; and numbers of partners per year of five for general MSM and eight for high-risk MSM.

We assumed that the high-risk MSM population comprised 20% of the general MSM population and that the initial HIV prevalence therein was nearly double that of general MSM^8–11^. Individuals entered the model at an age of 15 years and were followed for 20 years or until the age of 64 years. Mortality comprised HIV-related and non-HIV-related deaths.

We confirmed with the Institutional Review Board (IRB) of Severance Hospital that ethics approval was not needed before this study began since we did not utilize any personally identifiable information about human subjects.

### Parameters and values

Table S1 shows the parameter values for each of the variables, descriptions, and references. The values were decided by the best available local epidemiological data, literature reviews, investigator’s derivations, and calculations based on mathematical formulas. Demographic parameter values ($\rho$: entry rate, $\mu$: maturation rate, $\delta$: non-AIDS death rate) were based on Korea census data, and sexual behavior ($n$: number of partners), screening ($\psi$: fraction of test, ${\omega }_{id}$: average duration of identification), treatment ($\phi$: fraction of starting ART, $\widehat{\phi }$: rate of starting ART, $\gamma$: rate of discontinuing ART), health care costs, and medical costs related to the MSM and HIV patients were obtained from the Korea Centers for Disease Control & Prevention (KCDC) surveillance data and National Health Insurance Claims data^11–17^. These local epidemiological data described authenticity as demonstrated by references cited in the text. Other parameters related to disease transmission ($\pi$: transmission rate) and progression ($\theta$: progression rate, $\alpha$: AIDS death rate), sexual behavior ($u$: condom usage, $\kappa$: condom effectiveness), testing (${p}_{id}$: reduction of sexual behavior), drug efficacy (${\epsilon }_{pr}$: PrEP efficacy, ${\epsilon }_{tx}$: ART efficacy), and QALYs for each status, were taken by referring to the studies presented in Table S1.

We modeled HIV transmission via homosexual contact based on number of male sexual partners and average condom use^8,9,18^. The probability of HIV transmission between serodiscordant partners depended on the infected individual’s disease stage and ART use, as well as the PrEP status of the uninfected person^19–22^. HIV disease progression was based on the average time in each of four disease stages: acute infection, asymptomatic HIV, symptomatic HIV, and AIDS. Progression rates were based on HIV natural history and ART status^6,19,23,24^. We assumed that all persons with HIV were offered ART, given recent guidelines recommending ART initiation for all HIV-infected individuals regardless of CD4 cell counts^25^. The benefits of ART and suppression of viral replication included improved quality of life and reduced disease transmission, progression, and mortality^25^. We assumed a 99% reduction in sexual infectivity due to ART use for treatment of HIV infection^6,19,21,26–28^. To be treated, persons with HIV must be identified as infected. We assumed that 40% of MSM are currently screened annually using antibody tests. Accordingly, we assumed a 20% reduction in risky behavior for both infected and uninfected persons after HIV screening^19,29–31^.

### PrEP strategies

We considered two strategies: PrEP for the general MSM population and PrEP for high-risk MSM. We compared PrEP strategies to the status quo of no PrEP use. We assumed that MSM receiving PrEP would begin immediately and would remain on PrEP for the 20-year time horizon or until aging out of the model. We based our PrEP protocol on the Korean guidelines on PrEP use^32^. MSM were initiated on PrEP after a negative HIV antibody test, adequate calculated creatinine clearance, and testing for sexually transmitted infections. The PrEP regimen included daily TDF/FTC and physician visits four times per year, or every three months, at which time HIV-negative status was confirmed with an antibody test and risk-reduction counseling and condoms were provided. Additionally, sexually transmitted infection testing was performed every 6 months, and renal function was tested annually. Persons who became HIV infected while on PrEP were provided with appropriate counseling and discontinued PrEP once infection was detected. We assumed TDF/FTC reduces the probability of acquiring HIV by 80%, based on the overall reduction in incidence seen in previous study subjects^1,33–35^. PrEP was more effective in preventing HIV infection in iPrEx subgroups with higher adherence; therefore, we varied PrEP efficacy in sensitivity analysis. Although risk compensation is a concern with PrEP use, there is no evidence regarding the effect of PrEP on sexual risk^30^. Hence, in our base case, we assumed no change in risky behavior from counseling.

Various studies have assessed willingness and likelihood to use PrEP in surveyed MSM populations with varying conclusions^36–39^. The percentage of MSM who will ultimately use PrEP is unknown and will depend on numerous factors, such as public health campaigns and access to healthcare. We focused our results on 20%, 50%, and 100% of uninfected MSM initiating PrEP to illustrate differences in effectiveness and cost-effectiveness as the percentage of MSM on PrEP increases.

### Health outcomes and costs

We simulated the population over time and calculated discounted costs for each PrEP use scenario. The key parameters and values for cost and QALY are shown in Tables S2 and S3. We estimated quality of life for each health state and adjusted the utilities based on the average age of the modeled population. We assumed no reduction in quality of life from PrEP, as clinical trials have shown minimal side effects from TDF/FTC^1,32–35^. In sensitivity analysis, we evaluated the impact of decreased quality of life while on PrEP, as study participants on PrEP were more likely than those on placebo to experience minor side effects such as nausea^1,32–35^. We included costs associated with medical care in each health state, PrEP, and HIV testing and diagnosis to calculate total health-related costs. Baseline medical costs, HIV-related healthcare costs (with costs of associated co-morbidities), and cost of ART were estimated from the published literature^15,17,40^. Costs of antiretrovirals (ARVs) for PrEP were estimated as the average monthly wholesale price of TDF/FTC^14,15,17^. Costs of non-ARV components of the PrEP protocol and the HIV testing protocol were calculated according to the medical fee schedules of the Korea Health Insurance Review and Assessment Service, 2015. We also included discounted health-related costs and QALYs for the remaining lifetime of the population in the model at the end of the time horizon and for individuals who matured out of the modeled population.

## Results

### Current situation

**Figure 2:**
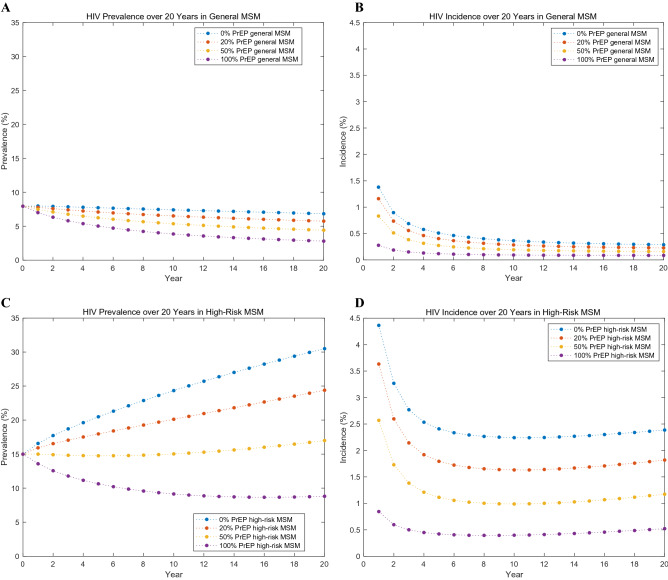
HIV prevalence and incidence over 20 years in general MSM (**A**,**B**) and high-risk MSM (**C**,**D**).

Our model projected that 17,130 and 14,485 new HIV infection will occur over 20 years for general MSM and high-risk MSM, respectively, if PrEP will not at all be implemented in MSM in South Korea (Table 1). This means that 84.6% of new HIV cases would arise from contact between high-risk individuals, despite high-risk MSM only accounting for 20% of all MSM in our model. The prevalence of HIV would remain mostly constant for 20 years in the general MSM population, but will increase to 30.5% in high-risk MSM (Table 1, Fig. 2).

**Table 1:** Benefits and costs of PrEP strategies over 20 years in general men who have sex with men (MSM) and high-risk MSM.

	HIV infection, New cases	HIV infection, Prevented cases	HIV prevalence at 20 years (%)	Incremental Costs (millions $)	Incremental QALYs	ICER relative to No PrEP ($/QALY)
**PrEP on general MSM**						
100% PrEP	4,250	12,880 (75.2%)	2.81	10,271	37,372	274,822
50% PrEP	9,612	7,518 (43.9%)	4.42	5,090	21,125	240,939
20% PrEP	13,812	3,318 (19.4%)	5.75	2,023	9,123	221,774
Status quo (No PrEP)	17,130		6.84			
**PrEP on high-risk MSM**						
100% PrEP	3,191	11,294 (78.0%)	8.81	1,509	29,242	51,597
50% PrEP	7,672	6,813 (47.0%)	17.0	701	17,450	40,200
20% PrEP	11,468	3,017 (20.8%)	24.4	266	7,739	34,346
Status quo (No PrEP)	14,485		30.5

### Prevented HIV cases by PrEP implementation

We projected the number of averted cases with new HIV infection by implementing PrEP in MSM population with different coverage rates (20%, 50%, and 100% of MSM population). Our prediction suggested that the number of prevented HIV infections by PrEP would reach 12,880 cases and 11,294 cases if PrEP were distributed to 100% of general MSM and high-risk MSM, respectively. Compared with no PrEP, there would be 75.2% and 78.0% reductions in new HIV cases among each group over 20 years (Table 1). Initiation of PrEP targeting high-risk MSM gives comparable numbers of averted cases with that in general MSM at the same proportion of PrEP introduction. This implies that similar numbers of new HIV cases can be averted by targeting high-risk MSM, with fewer numbers of MSM taking PrEP.

Over time, the prevalence of HIV infection for the general MSM slowly decreased, and at the end of the time horizon, there was a reduction of up to only 4% points. On the other hand, in the high-risk MSM population, the prevalence and incidence were greatly reduced, and in the case of 100% PrEP, the prevalence decreased by about 22%. However, to reduce the prevalence, all high-risk MSM need to receive PrEP (Table 1, Fig. 2).

### Cost-effectiveness analysis

**Figure 3:**
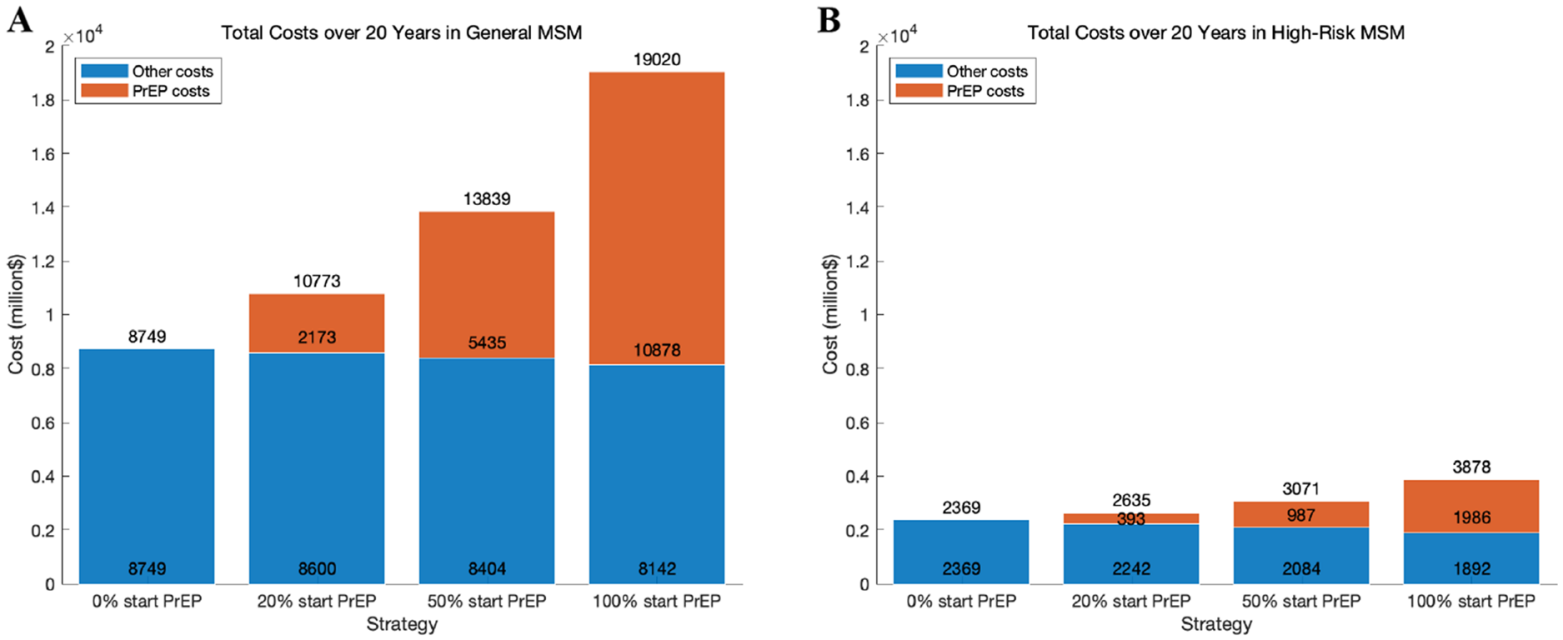
Total costs, consisting of PrEP costs and other costs, for each PrEP use scenario of targeting 0%, 20%, 50%, and 100% of general MSM (**A**) and high-risk MSM populations (**B**).

Considering the averted number of new infections, at the current price of TDF/FTC ($5,800/year), PrEP for general MSM population is predicted to gain 37,372 QALYs at a cost of $274,822 per QALY gained over 20 years relative to status quo. Initiating PrEP in high-risk MSM would improve the cost-effectiveness, adding 29,242 QALYs at a cost of $51,597 per QALY gained, within the Korea willingness-to-pay threshold of $56,000/QALY gained. In each scenario, implementing PrEP for 100% of the general and high-risk MSM population at current TDF/FTC costs would result in the PrEP cost exceeding the sum of the other costs (Fig. 3). Because PrEP targeting high-risk MSM reduces a similar number of new HIV cases with a fewer number of individuals initiating PrEP, compared with targeting the general MSM, there is a considerable reduction of additional costs due to PrEP relative to the reduction of QALY gains. Consequently, the ICERs under the willingness-to-pay threshold could be achieved for PrEP initiation in high-risk MSM (Table 1).

### Sensitivity analysis

**Figure 4:**
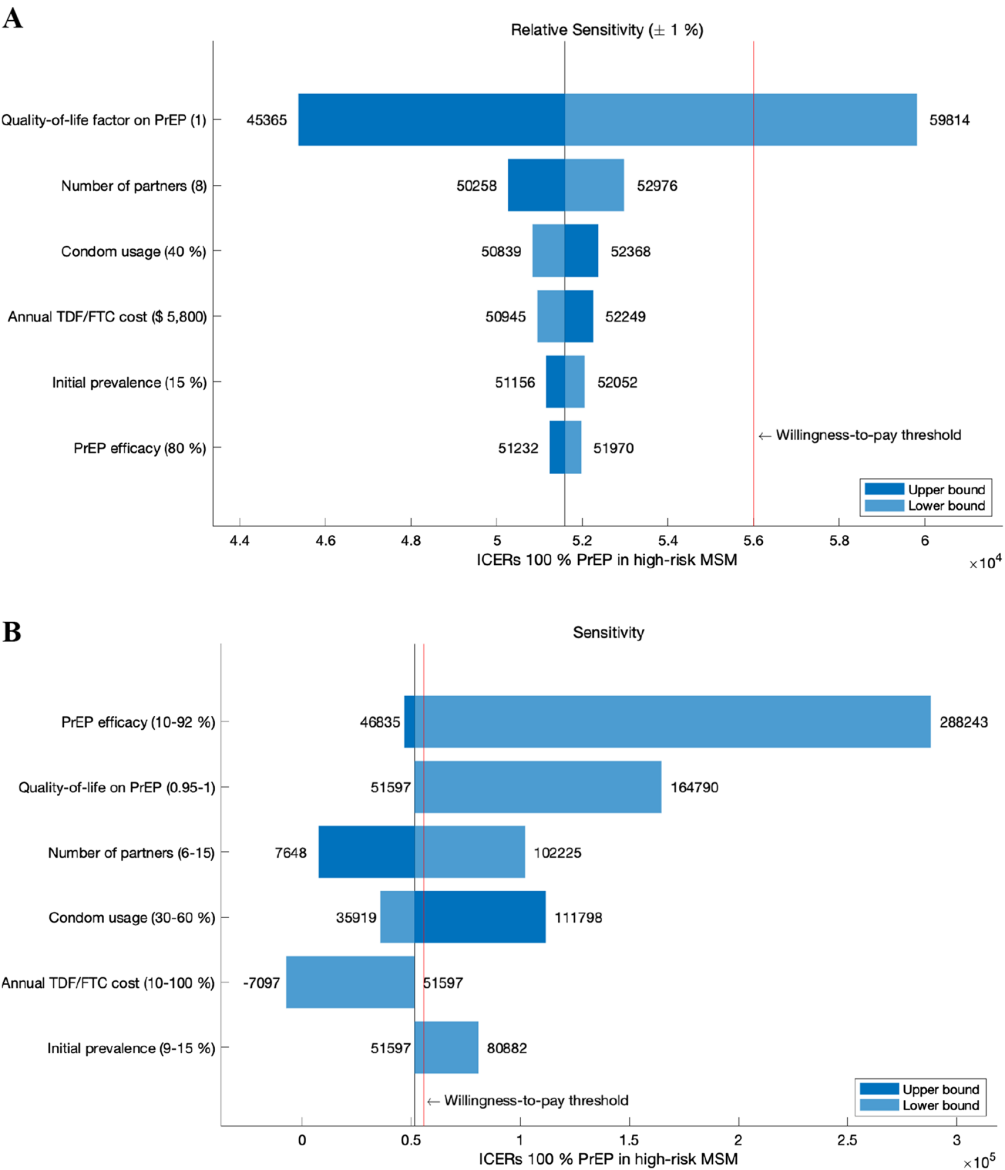
Tornado diagrams of ICERs with respect to the six most sensitive parameters at distributing PrEP for 100% of high-risk MSM. (**A**) Relative sensitivity between the parameters with perturbation of parameters by $\pm$ 1%. (**B**) Sensitivity by varying parameters within a feasible range.

We measured the sensitivity of ICER at distributing PrEP for 100% of high-risk MSM. First, all model parameters were perturbed $\pm$ 1% to compare the relative impact on ICERs among the parameters. Then, the sensitivity of the six most influential parameters were displayed in tornado diagrams (Fig. 4A). The cost-effectiveness of PrEP was highly sensitive to quality-of-life on PrEP, the number of partners, condom usage, annual PrEP cost, initial HIV prevalence, and PrEP efficacy. The quality-of-life factor for PrEP and TDF/FTC cost are significant because ICER directly depends on changes in QALYs and cost. Cost-effectiveness is considerably sensitive to the number of partners and condom usage. These are involved in the transmission rate of HIV, which plays a key role in infection dynamics. The initial prevalence of HIV determines the initial values of each compartment, and the results of the compartmental model are often sensitive to those values. Efficacy of PrEP took the sixth place as it indicates the degree of prevention achieved by the main control measure in the scenario.

While the analysis of relative sensitivity varying the parameters at the same level is standard theoretically, it may not be reasonable in practice because the feasible range of each parameter can differ greatly. In this context, we evaluated sensitivity by varying the six most influential parameters within a feasible range (Fig. 4B). If we assume lower PrEP efficacy ordiscount quality of life factor due to PrEP relative to current values, the ICERs will soar above the willingness-to-pay threshold. Assuming more partners or less condom usage would make PrEP initiation more effective. Lowering the price of TDF/FTC would be a sure way to get ICERs under the threshold, and cost savings with a negative ICER value can be achieved if the price is reduced 10% though it may be unrealizable. PrEP is less cost-effective if the initial prevalence in high-risk MSM is less than 15%.

**Figure 5:**
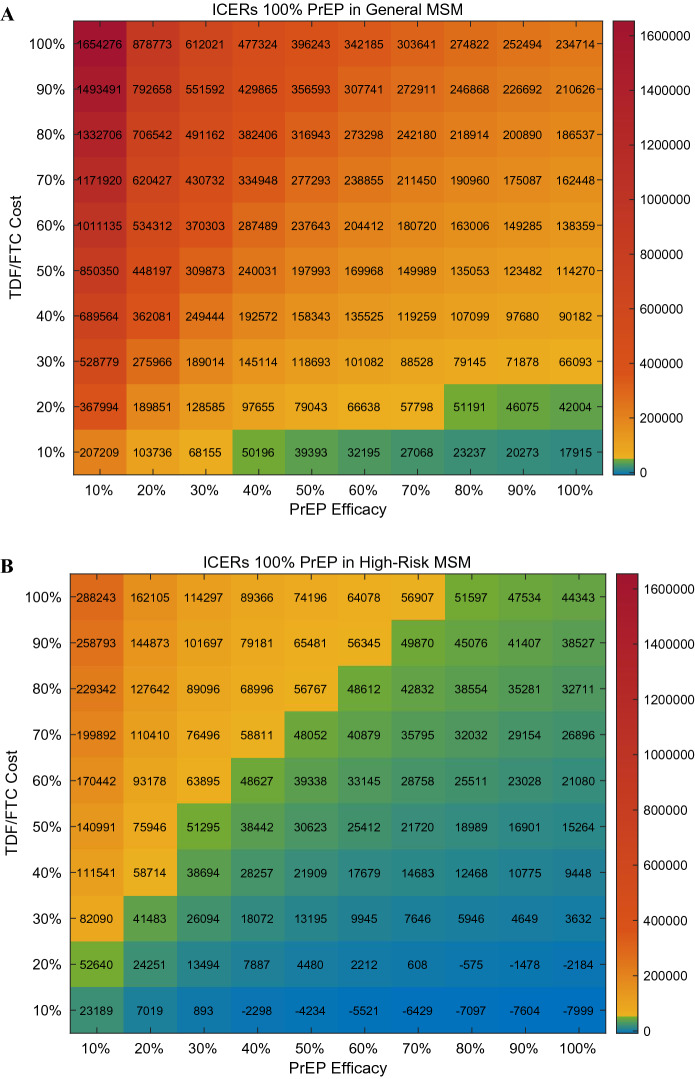
Heat maps of ICERs as a function of PrEP efficacy and PrEP cost in general MSM (**A**) and high-risk MSM (**B**).

**Figure 6:**
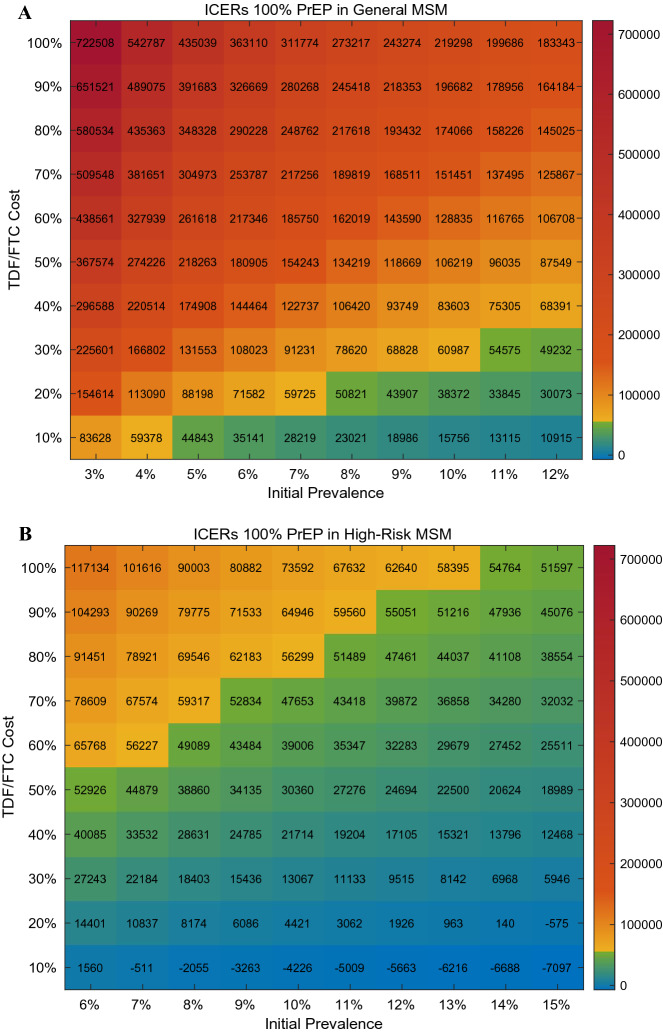
Heat maps of ICERs as a function of initial prevalence and PrEP cost in general MSM (**A**) and high-risk MSM (**B**).

Parameters of interest were selected among the six most sensitive parameters to perform two-way sensitivity analysis. The region of PrEP efficacy and TDF/FTC cost satisfying the willingness-to-pay threshold of $56,000/QALY gained is shown in heat maps (Fig. 5). With current PrEP efficacy (80%) and cost (100%), we can barely achieve cost-effectiveness by targeting PrEP to 100% of the high-risk MSM population. Reductions in the cost of TDF/FTC or improvements in PrEP efficacy would improve the cost-effectiveness of targeting PrEP to 100% of high-risk MSM. To achieve cost-effectiveness of 100% PrEP in general MSM, however, a 90% reduction in TDF/FTC cost is required, assuming PrEP efficacy is better than 40%. Two-way sensitivity was also analyzed in regards to region of initial prevalence of HIV infection and TDF/FTC cost to assess their combined impact on ICERs (Fig. 6). For the general MSM population, assuming a higher initial prevalence would not support the cost-effectiveness of PrEP unless the cost of TDF/FTC decreases to 10–20% of the current price. Meanwhile, 100% PrEP initiation in high-risk MSM would be cost-effective when the initial prevalence is higher than now. Even if it is lower than what we expect, ICERs lower than the willingness-to-pay threshold can be achieved by reducing the price of TDF/FTC up to 50%.

## Discussion

According to a report from the KCDC, the cumulative number of confirmed HIV infections was 17,500 as of December 2018, and the number of newly-infected cases per year was 1,206 in 2018^12^. Although the prevalence of confirmed HIV infections is still low in Korea (< 0.05%), the actual number of persons with HIV may be larger than reported by the Ministry, especially since the recent epidemiology of HIV infections in Korea is characterized by an increasing number of newly diagnosed HIV cases and proportion of late-presentation diagnoses^8,41–43^. Based on self-reports of sexual practices in 2015, MSM and heterosexual contact accounted for 44% and 56% of sexually transmitted cases, respectively. Given to the stigma attached to homosexuality within Korean society, the rate of MSM might be underestimated in the official report.

Despite efforts to overcome HIV, the number of newly diagnosed persons with HIV has continuously risen^12^. Multiple prevention measures have the possibility of impacting HIV incidence in South Korea, including early diagnosis, early treatment, and PrEP. A previous study investigated how each of these interventions could impact the local HIV epidemic, especially among MSM, who have become a major risk group in South Korea, using a mathematical model^7^. In a previous study, multiple prevention measures for HIV incidence in South Korea were considered, including early diagnosis, early treatment, and PrEP. The model simulation suggested that PrEP and early diagnosis could be effective at reducing HIV incidence in South Korea among MSM. However, the study did not consider cost-effectiveness of interventions, which would be important to help guide public health decisions^44^.

In our study, which is the first to estimate cost-effectiveness of PrEP in South Korea, we demonstrated that implementing PrEP in high-risk MSM (presumably 20% of MSM) would improve the cost-effectiveness, within the South Korea willingness-to-pay threshold of $56,000/QALY gained, and the cost-effectiveness of PrEP was highly sensitive to annual PrEP cost, quality-of-life on PrEP, and initial HIV prevalence. However, distributing PrEP for all MSM seems not to be cost-effective in South Korea. Total costs over 20 years for PrEP in all MSM was expected to be much higher than total costs for PrEP in high-risk MSM. Since the cost-effectiveness estimates were derived from parameter values of current epidemiology of South Korea, the estimates depend on epidemic changes, and it is unclear from the modeling whether increasing early diagnosis among MSM in South Korea would change these estimates.

Our findings in Korea that targeting PrEP to for high-risk MSM and any reduction in PrEP cost would improve cost-effectiveness in Korea are consistent with literature in other countries, including the United States and Australia^30,45–48^. Several of previous studies using mathematical models have shown the necessity of targeting PrEP to MSM at high risk of HIV infection for PrEP to be cost-effective^30,45–47^. Another study, which focused on a targeted PrEP strategy, found diminishing cost-effectiveness ratios with larger proportions of high-risk MSM on PrEP^48^. Considering the willingness-to-pay threshold in South Korea, it was barely cost-effective by applying PrEP only to high-risk MSM population.

The cost-effectiveness of PrEP was highly sensitive to annual PrEP cost, and a price reduction could decrease the cost-effectiveness ratio. In South Korea, generic ARVs are not available, and the PrEP cost for this study was calculated with the price of original TDF/FTC. Given that the patent for TDF/FTC will expire, price reductions can be expected in future. In addition, we do not know the future prices of ARVs for treatment of HIV infection. The prices of future ARVs might affect our results.

This study has several limitations. First, our model was not stratified by the level of HIV infection risk. To take this into the model, data regarding contact patterns between heterogeneous risk groups are required for the calibration of the model. No reliable sources about sexual behaviors between the risk groups are available in South Korea, however, future studies should consider surveys to quantify contact patterns. Second, we did not include an on-demand PrEP strategy in our model. A previous study demonstrated the effect of on-demand PrEP for high-risk MSM^49^. Given the higher cost of daily PrEP, the cost-effectiveness ratio of on-demand PrEP might increase. Third, our results might be limited, and our estimates might also be biased because we included parameter estimates from Western countries, as with most mathematical models. Data from other countries could influence the conclusions of our study. Fourth, our derivations of some parameters were not validated, although they were based on the best available epidemiologic data. For example, we estimated the initial prevalence of MSM by analyzing HIV incidence, sex ratio, and results in the literature. Also, no change in risk behavior after PrEP was assumed. It has, however, been reported that its impact on PrEP effectiveness is not significant by a previous study^7^. Fifth, improvements in HIV care cascade towards UNAIDS 90–90–90 goal and new therapeutics including HIV vaccine have not been considered over the next 20 years, because it is not feasible to quantify the impact of those on HIV incidence. Consequently, the observed effectiveness of PrEP may involve overestimation of the prevalence of HIV.

## Conclusion

Initiating PrEP in a larger proportion of MSM in Korea would prevent more HIV infections, although at an increasing cost per QALY gained. PrEP only in high-risk MSM and reductions in the drug costs for PrEP would improve cost-effectiveness.

## Supplementary information

Supplementary Information
