# Supplementary material for: Cost-effectiveness analysis of pre-exposure prophylaxis for the prevention of HIV in men who have sex with men in South Korea: a mathematical modelling study
Source: Sci Rep. 2020 Sep 3;10:14609. doi: 10.1038/s41598-020-71565-y (PMC7471951; doi:10.1038/s41598-020-71565-y)
Supplement: Supplementary file 1 — Supplementary Information [file 41598_2020_71565_MOESM1_ESM.docx]

**Cost-effectiveness Analysis of Pre-Exposure Prophylaxis for the Prevention of HIV in Men Who Have Sex with Men in South Korea: a Mathematical Modelling Study**

Heun Choi^1,*^, Jiyeon Suh^2,*^, Woonji Lee^1^, Jun Hyoung Kim^1^, Jung Ho Kim^1^, Hye Seong^1^, Jin Young Ahn^1^, Su Jin Jeong^1^, Nam Su Ku^1^, Yoon Soo Park^1^, Joon Sup Yeom^1^, Changsoo Kim^3^, Hee-Dae Kwon^4^, Davey M. Smith^5,6^, Jeehyun Lee^2,7^, Jun Yong Choi^1,#^

^1^Department of Internal Medicine and AIDS Research Institute, Yonsei University College of Medicine, Seoul, Republic of Korea

^2^Department of Computational Science and Engineering, Yonsei University, Seoul, Republic of Korea

^3^Department of Preventive Medicine, Yonsei University College of Medicine, Seoul, Republic of Korea

^4^Department of Mathematics, Inha University, Incheon, Republic of Korea

^5^Department of Medicine, University of California San Diego, La Jolla, California, United States of America

^6^Veterans Affairs San Diego Healthcare System, San Diego, California, United States of America

^7^Department of Mathematics, Yonsei University, Seoul, Republic of Korea

*Heun Choi and Jiyeon Suh equally contributed to this study.

#Correspondence should be addressed to Jun Yong Choi, MD, PhD (E-mail: SERAN@yuhs.ac)

**Supplementary materials> Transmission equations, parameters, costs, and quality of life factors for this study**

Figure S1. Transmission equations.

NOTE. See table S1 for referring the meaning of each abbreviations

$$\frac{dX_{1}}{dt}=\rho\sum_{i} X_{i}-\left( \sum_{2<j<15,j>15} \beta_{1,j}\left( t \right) \right)X_{1}-\psi X_{1}+\omega_{id}X_{2}-\left( \mu_{1}+\delta_{1} \right)X_{1}$$

$$\frac{dX_{2}}{dt}=-\left( \sum_{2<j<15,j>15} \beta_{2,j}\left( t \right) \right)X_{2}+\psi X_{1}-\omega_{id}X_{2}+\omega_{pr}X_{15}-\left( \mu_{2}+\delta_{2} \right)X_{2}$$

$$\frac{dX_{3}}{dt}=\left( \sum_{2<j<15,j>15} \beta_{1,j}\left( t \right) \right)X_{1}+\left( \sum_{2<j<15,j>15} \beta_{2,j}\left( t \right) \right)X_{2}-\left( \psi+\nu_{3} \right)X_{3}-\theta_{3}X_{3}-\left( \mu_{3}+\delta_{3} \right)X_{3}$$

$$\frac{dX_{4}}{dt}=\left( \psi+\nu_{3} \right)(1-\phi_{3})X_{3}-\theta_{4}X_{4}-\hat{\phi}_{4}X_{4}+\gamma_{11}X_{11}+\omega_{m}X_{16}-\left( \mu_{4}+\delta_{4} \right)X_{4}$$

$$\frac{dX_{5}}{dt}=\theta_{3}X_{3}-\left( \psi+\nu_{5} \right)X_{5}-\theta_{5}X_{5}-\left( \mu_{5}+\delta_{5} \right)X_{5}$$

$$\frac{dX_{6}}{dt}=\theta_{4}X_{4}+\left( \psi+\nu_{5} \right)(1-\phi_{5})X_{5}-\theta_{6}X_{6}-\hat{\phi}_{6}X_{6}+\gamma_{12}X_{12}+\omega_{m}X_{17}-\left( \mu_{6}+\delta_{6} \right)X_{6}$$

$$\frac{dX_{7}}{dt}=\theta_{5}X_{5}-\left( \psi+\nu_{7} \right)X_{7}-\theta_{7}X_{7}-\left( \mu_{7}+\delta_{7} \right)X_{7}$$

$$\frac{dX_{8}}{dt}=\theta_{6}X_{6}+\left( \psi+\nu_{7} \right)(1-\phi_{7})X_{7}-\theta_{8}X_{8}-\hat{\phi}_{8}X_{8}+\gamma_{13}X_{13}-\left( \mu_{8}+\delta_{8} \right)X_{8}$$

$$\frac{dX_{9}}{dt}=\theta_{7}X_{7}-\left( \psi+\nu_{9} \right)X_{9}-\left( \mu_{9}+\delta_{9}+\alpha_{9} \right)X_{9}$$

$$\frac{dX_{10}}{dt}=\theta_{8}X_{8}+\left( \psi+\nu_{9} \right)\left( 1-\phi_{9} \right)X_{9}-\hat{\phi}_{10}X_{10}+\gamma_{14}X_{14}-\left( \mu_{10}+\delta_{10}+\alpha_{10} \right)X_{10}$$

$$\frac{dX_{11}}{dt}=\left( \psi+\nu_{3} \right)\phi_{3}X_{3}+\hat{\phi}_{4}X_{4}-\gamma_{11}X_{11}-\theta_{11}X_{11}-\left( \mu_{11}+\delta_{11} \right)X_{11}$$

$$\frac{dX_{12}}{dt}=\left( \psi+\nu_{5} \right)\phi_{5}X_{5}+\theta_{11}X_{11}+\hat{\phi}_{6}X_{6}-\gamma_{12}X_{12}-\theta_{12}X_{12}-\left( \mu_{12}+\delta_{12} \right)X_{12}$$

$$\frac{dX_{13}}{dt}=\left( \psi+\nu_{7} \right)\phi_{7}X_{7}+\theta_{12}X_{12}+\hat{\phi}_{8}X_{8}-\gamma_{13}X_{13}-\theta_{13}X_{13}-\left( \mu_{13}+\delta_{13} \right)X_{13}$$

$$\frac{dX_{14}}{dt}=\left( \psi+\nu_{9} \right)\phi_{9}X_{9}+\theta_{13}X_{13}+\hat{\phi}_{10}X_{10}-\gamma_{14}X_{14}-\left( \mu_{14}+\delta_{14}+\alpha_{14} \right)X_{14}$$

$$\frac{dX_{15}}{dt}=-\left( \sum_{2<j<15,j>15} \beta_{15,j}\left( t \right) \right)X_{15}-\omega_{pr}X_{15}-\left( \mu_{15}+\delta_{15} \right)X_{15}$$

$$\frac{dX_{16}}{dt}=\left( \sum_{2<j<15,j>15} \beta_{15,j}\left( t \right) \right)X_{15}-\theta_{16}X_{16}-\omega_{m}X_{16}-\left( \mu_{16}+\delta_{16} \right)X_{16}$$

$$\frac{dX_{17}}{dt}=\theta_{16}X_{16}-\omega_{m}X_{17}-\left( \mu_{17}+\delta_{17} \right)X_{17}$$

Table S1. Key Model Parameters

| Parameter | Description | Value | Range | Reference | Assumptions |
| --- | --- | --- | --- | --- | --- |
| Initial Values |  |  |  |  |  |
| $\sum_{i} X_{i}(t=0)$ | Initial total MSM population age 15-64 | 217,280 | 200,000-250,000 | [1, 2] |  |
|  | Initial HIV prevalence in MSM (%) | 7.95% (general)  15% (high-risk) | 3.5-15% | [3-6] |  |
|  | Awareness of HIV status (%) | 60% | 40%-80% | [7] |  |
| $X_{1}$ | Number of uninfected-unaware MSM HIV | 80,000 | 65,000-100,000 | [7-9] | 40% of HIV-uninfected MSM |
| $X_{2}$ | Number of uninfected-aware MSM HIV | 120,000 | 100,000-142,000 | [7-9] | 60% of HIV-uninfected MSM |
| $X_{3}$ | Number of acute HIV-unaware MSM | 800 | 400-3,000 | [7-9] |  |
| $X_{4}$ | Number of acute HIV-aware MSM | 500 | 250-2,000 | [7-9] |  |
| $X_{5}$ | Number of asymptomatic HIV-unaware MSM | 2,400 | 1,200-9,000 | [7-9] |  |
| $X_{6}$ | Number of asymptomatic HIV-aware MSM | 600 | 300-2,500 | [7-9] |  |
| $X_{7}$ | Number of symptomatic HIV-unaware MSM | 1,800 | 900-6,300 | [9] |  |
| $X_{8}$ | Number of symptomatic HIV-aware MSM | 1,200 | 600-4,200 | [9] |  |
| $X_{9}$ | Number of AIDS-unaware MSM | 2,000 | 1,000-7,000 | [9] |  |
| $X_{10}$ | Number of AIDS-aware MSM | 3,000 | 1,500-10,500 | [9] |  |
| $X_{11}$ | Number of acute HIV-treated with ART MSM | 470 | 250-2,000 | [6, 10, 11] | 94% of acute HIV-aware MSM |
| $X_{12}$ | Number of asymptomatic HIV-treated with ART MSM | 560 | 300-2,500 | [6, 10, 11] | 94% of asymptomatic HIV-aware MSM |
| $X_{13}$ | Number of symptomatic HIV-treated with ART MSM | 1,130 | 600-4,200 | [6, 10, 11] | 94% of symptomatic HIV-aware MSM |
| $X_{14}$ | Number of AIDS-treated with ART MSM | 2,820 | 1,500-10,500 | [6, 10, 11] | 94% of HIV-aware AIDS MSM |
| $X_{15}$ | Number of uninfected-PrEP MSM |  |  |  | 20% or 40% or 60% of HIV-uninfected MSM |
| $X_{16}$ | Number of acute HIV-PrEP MSM |  |  |  | 20% or 40% or 60% of acute HIV-infected MSM |
| $X_{17}$ | Number of asymptomatic HIV-PrEP MSM |  |  |  | 20% or 40% or 60% of asymptomatic HIV-infected MSM |
| Demographic Parameters |  |  |  |  |  |
| $\rho$ | Entry rate of individuals into the model at age 15 | 0.0175 | 0.01-0.03 | [1] |  |
| $\mu_{i}$ | Maturation rate out of compartment $i$ at age 64 | 0.0109 | 0.005-0.025 | [1] |  |
| $\delta_{i}$ | Non-AIDS death rate for compartment $i$ | 0.0127 |  | [1] |  |
| Sexual Behavior Parameters |  |  |  |  |  |
| $n_{i}$ | Annual number of male partners of individuals in compartment $i$ | 5 (general)  8 (high-risk) | 2-15 | [3, 7] |  |
| $u_{i}$ | Condom usage with male partners by individuals in compartment $i$ (%) | 40% | 30-60% | [3, 4, 7] |  |
| $\kappa$ | Condom effectiveness in reducing HIV transmission (%) | 90% | 85-95% | [12] |  |
| Transmission Parameters |  |  |  |  |  |
| $\text{π}_{\text{k}}$ | Annual probability of HIV transmission per unprotected sexual partnership between an uninfected male and an infected male with HIV status k |  |  |  |  |
| $\pi_{Acute HIV}$ | Probability of HIV transmission per unprotected sexual partnership of MSM with acute HIV | 0.210 | 0.10-0.40 | [13-15] |  |
| $\pi_{Asymptomatic HIV}$ | Probability of HIV transmission per unprotected sexual partnership of MSM with asymptomatic HIV | 0.039 | 0.02-0.08 | [13-16] |  |
| $\pi_{Symptomatic HIV}$ | Probability of HIV transmission per unprotected sexual partnership of MSM with symptomatic HIV | 0.039 | 0.02-0.08 | [13-16] |  |
| $\pi_{\mathrm{AIDS}}$ | Probability of HIV transmission per unprotected sexual partnership of MSM with AIDS | 0.160 | 0.08-0.30 | [13-16] |  |
| $\sigma_{i,j}$ | Annual probability of HIV transmission per unprotected sexual partnership between uninfected (compartment $i$, $i$ = 1, 2, 13) and infected (compartment $j$, $j$=3-12, 14-15) individuals | $\sigma_{i,j}=\pi_{j}\left( 1-\epsilon_{tx} \right)(1-\epsilon_{pr})$ | |  |  |
| $\beta_{i,j}(t)$ | Sufficient contact rate at time t between uninfected (compartment $i$, $i$ = 1, 2, 13) and infected (compartment $j$, $j$=3-12, 14-15) individuals | $\beta_{i,j}\left( t \right)= 1-\left( 1-P\left( select \right)\sigma_{i,j} \right)^{n_{i}(1-u_{i}\kappa)}$  $P\left( select \right)= \frac{X_{j}\left( t \right)n_{j}(1-u_{j}\kappa)}{\sum_{k} X_{k}\left( t \right)n_{k}(1-u_{k}\kappa)}$ | |  |  |
| Progression Parameters |  |  |  |  |  |
| $\theta_{i}$ | HIV disease progression rate for compartment $i$ ($i$ = 3-8, 11-13, 16) |  |  |  |  |
| $1/{\theta_{3,4,16}}$ | Average disease duration (years) of acute HIV without ART | 0.25 | 0.08-0.40 | [17-21] |  |
| $1/{\theta_{11}}$ | Average disease duration (years) of acute HIV treated with ART | 0.25 | 0.08-0.40 |  |  |
| $1/{\theta_{5,6}}$ | Average disease duration (years) of asymptomatic HIV without ART | 7 | 6-10 |  |  |
| $1/{\theta_{12}}$ | Average disease duration (years) of asymptomatic HIV treated with ART | 30 | 20-40 |  |  |
| $1/{\theta_{7,8}}$ | Average disease duration (years) of symptomatic HIV without ART | 3 | 1-4 | [16, 22-24] |  |
| $1/{\theta_{13}}$ | Average disease duration (years) of symptomatic HIV treated with ART | 18 | 12-30 | [16, 24-26] |  |
| $\alpha_{i}$ | AIDS death rate for compartment $i$ ($i$ =9, 10, 14) |  |  |  |  |
| $1/{\alpha_{9,10}}$ | Average disease duration (years) of AIDS without ART | 2 | 1-3 | [16, 22-24] |  |
| $1/{\alpha_{14}}$ | Average disease duration (years) of AIDS treated with ART | 5 | 2-15 | [16, 24-26] |  |
| Testing Parameters |  |  |  |  |  |
| $\psi$ | Fraction of population tested annually or probability of screening (%) | 40% | 20-60% | [7] |  |
| $\nu_{i}$ | Probability of symptomatic testing or case-detection for individuals in compartment $i$ ($i$ = 3, 5, 7, 9) annually (%) |  |  | Assumed |  |
| $\nu_{3}$ | Annual probability of case finding acute HIV  (%) | 40% | 20-60% |  |  |
| $\nu_{5}$ | Annual probability of case finding asymptomatic HIV (%) | 20% | 0-40% |  |  |
| $\nu_{7}$ | Annual probability of symptom-based case finding symptomatic HIV (%) | 40% | 20-60% |  |  |
| $\nu_{9}$ | Annual probability of symptom-based case finding AIDS (%) | 60% | 40-80% |  |  |
| $1/{\omega_{id}}$ | Average duration of identification status for uninfected individuals (years) | 2 | 0.5-8 | Assumed |  |
| $p_{id}$ | Reduction in sexual behavior (number of male partners) due to screening and counseling (%) | 20% | 0-50% | [16, 27-29] |  |
|  | Sensitivity of antibody test, post-seroconversion (%) | 99.5% | 98-99.9% | [30-33] |  |
|  | Specificity of antibody test, post-seroconversion (%) | 99.9994% | 99-100% | [30-33] |  |
| Treatment Parameters |  |  |  |  |  |
| $\epsilon_{tx}$ | ART efficacy in reducing sexual infectivity in infected individuals | 99% | 90-100% | [14, 16, 24, 30, 34, 35] |  |
| $\phi_{i}$ | Fraction of individuals in compartment $i$ ($i$ = 3, 5, 7, 9) starting ART |  |  | [8-11] |  |
| $\phi_{3}$ | Fraction of individuals in compartment 3 starting ART | 30% |  |  |  |
| $\phi_{5}$ | Fraction of individuals in compartment 5 starting ART | 30% |  |  |  |
| $\phi_{7}$ | Fraction of individuals in compartment 7 starting ART | 80% | 70-90% |  |  |
| $\phi_{9}$ | Fraction of individuals in compartment 9 starting ART | 80% | 70-90% |  |  |
| $\hat{\phi}_{i}$ | Rate of individuals in compartment $i$ ($i$ = 4, 6, 8, 10) starting ART |  |  |  |  |
| ${1/\hat{\phi}}_{i}$ | Average duration (years) of stay in compartment $i$ ($i$ = 4, 6, 8, 10) before ART | 4 | 3-5 | [9, 36] |  |
| $\gamma_{i}$ | Rate of individuals in compartment $i$ ($i$ = 11, 12, 13, 14) discontinuing ART |  |  |  |  |
| $1/\gamma_{i}$ | Average duration (years) of stay in compartment $i$ ($i$ = 11, 12, 13, 14) before discontinuing ART | 20 | 10-30 | Assumed |  |
| PrEP Parameters |  |  |  |  |  |
| $\epsilon_{pr}$ | PrEP efficacy in reducing the probability of an uninfected individual acquiring HIV from homosexual contact with an infected individual  (%) | 80% | 10-92% | [37-40] |  |
| $pr_{i}$ | Fraction of individual in compartment $i$ starting PrEP ($i$ = 1,2,3) (%) |  | 0-100% |  |  |
| $1/{\omega_{pr}}$ | Average duration on PrEP (years) | 20 | 1-20 | Assumed |  |
| $1/{\omega_{m}}$ | Average time between PrEP monitoring visits (years) | 0.25 | 0.25-1 | [41] |  |
| $p_{pr}$ | Change in number of sexual partners due to PrEP (%) | 0% | -20-20% | Assumed |  |
| $c_{pr}$ | Change in condom use due to PrEP (%) | 0% | -20-20% | Assumed |  |

Table S2. Costs for this study

| Description | Value | Range | Reference |
| --- | --- | --- | --- |
| Annual HIV-related healthcare costs excluding ART costs |  |  |  |
| - Acute HIV – Untreated | 1,600 | 1,100-2,100 | Calculated [10, 11] |
| - Acute HIV – Treated with ART (exclude ART costs) | 1,600 | 1,100-2,100 |  |
| - Asymptomatic HIV – Untreated | 1,800 | 1,300-2,300 |  |
| - Asymptomatic HIV – Treated with ART (exclude ART costs) | 1,800 | 1,300-2,300 |  |
| - Symptomatic HIV – Untreated | 5,400 | 5,000-5,500 |  |
| - Symptomatic HIV – Treated with ART (exclude ART costs) | 3,600 | 3,000-4,000 |  |
| - AIDS – Untreated | 21,200 | 15,000-25,000 |  |
| - AIDS – Treated with ART (exclude ART costs) | 10,600 | 10,000-20,000 |  |
| Annual non-HIV-related healthcare costs for uninfected and infected individuals | 21,200 | 15,000-25,000 | [42] |
| Annual cost of ART | 7,100 | 5,500-10,000 | [10, 11, 43] |
| Annual cost of PrEP |  |  | [10, 11, 43] |
| - TDF/FTC | 5,800 |  |  |
| - STI testing | 200 |  |  |
| - Blood urea nitrogen and serum creatinine testing | 20 |  |  |
| - Physician visit | 50 |  |  |
| Cost of HIV testing |  |  | [10, 11, 43] |
| - Testing for HIV-uninfected population | 25 |  |  |
| - Testing for HIV-infected population | 25 |  |  |
| Cost of HIV diagnosis | 1,000 | 900-1,500 | [10, 11, 43] |
| Annual discount rate | 3% | 0-5% | [44] |
| Exchange rate | 1130.85 |  | [45] |

Table S3. Quality-of-Life Factors for this study

| Description | Value | Range | Source |
| --- | --- | --- | --- |
| Uninfected – No PrEP | 1.00 |  | [16, 46] |
| Uninfected – Receiving PrEP | 1.00 | 0.90-1.00 | [37] |
| Acute HIV – Unidentified | 0.92 | 0.73-0.97 | Calculated [18, 47-49] |
| Acute HIV – Identified | 0.86 | 0.68-0.91 | Calculated [18, 30, 47-50] |
| Acute HIV – Treated with ART | 0.93 |  |  |
| Asymptomatic – Unidentified | 0.91 | 0.85-0.95 | [30, 50] |
| Asymptomatic – Identified | 0.84 | 0.84-0.95 | [30] |
| Asymptomatic – Treated with ART | 0.92 |  |  |
| Symptomatic – Unidentified | 0.79 | 0.70-0.80 | [30, 51, 52] |
| Symptomatic – Identified | 0.72 | 0.70-0.80 | [50] |
| Symptomatic – Treated with ART | 0.83 | 0.82-0.87 | [16, 30, 51] |
| AIDS – Unidentified | 0.72 | 0.60-0.75 | [16, 30] |
| AIDS – Identified | 0.72 | 0.60-0.75 | [16, 51, 52] |
| AIDS – Treated with ART | 0.82 | 0.82-0.87 | [16, 51] |

**References**

1. Korean Statistical Information Service Vital Statistics of Korea <http://kosis.kr/statHtml/statHtml.do?orgId=101&tblId=DT_1IN1503&conn_path=I2> (2016).

2. Korea Centers for Disease Control and Prevention National Survey on the Sexual Consciousness <http://www.ndsl.kr/ndsl/search/detail/report/reportSearchResultDetail.do?cn=TRKO201600015992> (2015).

3. Sohn, A. & Cho, B. Knowledge, Attitudes, and Sexual Behaviors in HIV/AIDS and Predictors Affecting Condom Use among Men Who Have Sex with Men in South Korea. *Osong Public Health Res Perspect* **3**, 156-164 (2012).

4. Jung, M., Lee, J., Kwon, D. S. & Park, B. J. Comparison of sexual risky factors of men who have sex with men and sex-buying men as groups vulnerable to sexually transmitted diseases. *J Prev Med Public Health* **45**, 156-163 (2012).

5. Suguimoto, S. P. *et al.* Changing patterns of HIV epidemic in 30 years in East Asia. *Curr HIV/AIDS Rep* **11**, 134-145 (2014).

6. Korea Centers for Disease Control and Prevention Annual Report on the Notified HIV/AIDS in Korea 2017 <http://www.cdc.go.kr/menu.es?mid=a20301070305> (2018).

7. Sohn, A., Cho, B. & Kennedy, H. A. Identifying Barriers to Human Immunodeficiency Virus Testing for Men Who Have Sex with Men in South Korea. *Osong Public Health Res Perspect* **6**, 192-200 (2015).

8. Lee, J. H. *et al.* Increasing late diagnosis in HIV infection in South Korea: 2000-2007. *BMC Public Health* **10**, 411 (2010).

9. Kim, M. J. *et al.* Trend of CD4+ Cell Counts at Diagnosis and Initiation of Highly Active Antiretroviral Therapy (HAART): Korea HIV/AIDS Cohort Study, 1992-2015. *Infect Chemother* **49**, 101-108 (2017).

10. Korea Centers for Disease Control and Prevention Utilization of health care services among people with HIV infection <http://www.ndsl.kr/ndsl/search/detail/report/reportSearchResultDetail.do?cn=TRKO201600015993> (2015).

11. Korean Statistical Information Service Benefits by Classification of Disease Categories <http://kosis.kr/statHtml/statHtml.do?orgId=350&tblId=TX_35001_A061&conn_path=I2> (2015).

12. Long, E. F. *et al.* Effectiveness and cost-effectiveness of strategies to expand antiretroviral therapy in St. Petersburg, Russia. *Aids* **20**, 2207-2215 (2006).

13. Xiridou, M., Geskus, R., De Wit, J., Coutinho, R. & Kretzschmar, M. The contribution of steady and casual partnerships to the incidence of HIV infection among homosexual men in Amsterdam. *Aids* **17**, 1029-1038 (2003).

14. McCormick, A. W. *et al.* The effect of antiretroviral therapy on secondary transmission of HIV among men who have sex with men. *Clin Infect Dis* **44**, 1115-1122 (2007).

15. Wawer, M. J. *et al.* Rates of HIV-1 transmission per coital act, by stage of HIV-1 infection, in Rakai, Uganda. *J Infect Dis* **191**, 1403-1409 (2005).

16. Long, E. F., Brandeau, M. L. & Owens, D. K. Potential population health outcomes and expenditures of HIV vaccination strategies in the United States. *Vaccine* **27**, 5402-5410 (2009).

17. Pilcher, C. D. *et al.* Brief but efficient: acute HIV infection and the sexual transmission of HIV. *J Infect Dis* **189**, 1785-1792 (2004).

18. Vergis, E. N. & Mellors, J. W. Natural history of HIV-1 infection. *Infect Dis Clin North Am* **14**, 809-825, v-vi (2000).

19. Hollingsworth, T. D., Anderson, R. M. & Fraser, C. HIV-1 transmission, by stage of infection. *J Infect Dis* **198**, 687-693 (2008).

20. Pinkerton, S. D. How many sexually-acquired HIV infections in the USA are due to acute-phase HIV transmission? *Aids* **21**, 1625-1629 (2007).

21. Xiridou, M., Geskus, R., de Wit, J., Coutinho, R. & Kretzschmar, M. Primary HIV infection as source of HIV transmission within steady and casual partnerships among homosexual men. *Aids* **18**, 1311-1320 (2004).

22. Mellors, J. W. *et al.* Plasma viral load and CD4+ lymphocytes as prognostic markers of HIV-1 infection. *Ann Intern Med* **126**, 946-954 (1997).

23. Dunn, D. *et al.* Current CD4 cell count and the short-term risk of AIDS and death before the availability of effective antiretroviral therapy in HIV-infected children and adults. *J Infect Dis* **197**, 398-404 (2008).

24. Long, E. F., Brandeau, M. L. & Owens, D. K. The cost-effectiveness and population outcomes of expanded HIV screening and antiretroviral treatment in the United States. *Ann Intern Med* **153**, 778-789 (2010).

25. May, M. *et al.* Prognosis of HIV-1-infected patients up to 5 years after initiation of HAART: collaborative analysis of prospective studies. *Aids* **21**, 1185-1197 (2007).

26. Life expectancy of individuals on combination antiretroviral therapy in high-income countries: a collaborative analysis of 14 cohort studies. *Lancet* **372**, 293-299 (2008).

27. Rose, C. D. *et al.* HIV intervention for providers study: a randomized controlled trial of a clinician-delivered HIV risk-reduction intervention for HIV-positive people. *J Acquir Immune Defic Syndr* **55**, 572-581 (2010).

28. Juusola, J. L., Brandeau, M. L., Owens, D. K. & Bendavid, E. The cost-effectiveness of preexposure prophylaxis for HIV prevention in the United States in men who have sex with men. *Ann Intern Med* **156**, 541-550 (2012).

29. Chang, H. H. *et al.* Awareness and Acceptance of HIV Pre-exposure Prophylaxis among Medical Personnel and Men Who Have Sex with Men in Korea. *J Korean Med Sci* **33**, e91 (2018).

30. Sanders, G. D. *et al.* Cost-effectiveness of screening for HIV in the era of highly active antiretroviral therapy. *N Engl J Med* **352**, 570-585 (2005).

31. Owens, D. K., Nease, R. F., Jr. & Harris, R. A. Cost-effectiveness of HIV screening in acute care settings. *Arch Intern Med* **156**, 394-404 (1996).

32. Mylonakis, E., Paliou, M., Lally, M., Flanigan, T. P. & Rich, J. D. Laboratory testing for infection with the human immunodeficiency virus: established and novel approaches. *Am J Med* **109**, 568-576 (2000).

33. Greenwald, J. L., Burstein, G. R., Pincus, J. & Branson, B. A rapid review of rapid HIV antibody tests. *Curr Infect Dis Rep* **8**, 125-131 (2006).

34. Cohen, M. S. *et al.* Antiretroviral Therapy for the Prevention of HIV-1 Transmission. *The New England journal of medicine* **375**, 830-839 (2016).

35. Wilson, D. P., Law, M. G., Grulich, A. E., Cooper, D. A. & Kaldor, J. M. Relation between HIV viral load and infectiousness: a model-based analysis. *Lancet* **372**, 314-320 (2008).

36. Lee, S. *et al.* Predictors of Poor Retention in Care of HIV-infected Patients Receiving Antiretroviral Therapy in Korea: Five-Year Hospital-based Retrospective Cohort Study. *J Korean Med Sci* **31**, 376-381 (2016).

37. Grant, R. M. *et al.* Preexposure chemoprophylaxis for HIV prevention in men who have sex with men. *N Engl J Med* **363**, 2587-2599 (2010).

38. Fonner, V. A. *et al.* Effectiveness and safety of oral HIV preexposure prophylaxis for all populations. *Aids* **30**, 1973-1983 (2016).

39. Volk, J. E. *et al.* No New HIV Infections With Increasing Use of HIV Preexposure Prophylaxis in a Clinical Practice Setting. *Clin Infect Dis* **61**, 1601-1603 (2015).

40. McCormack, S. *et al.* Pre-exposure prophylaxis to prevent the acquisition of HIV-1 infection (PROUD): effectiveness results from the pilot phase of a pragmatic open-label randomised trial. *Lancet* **387**, 53-60 (2016).

41. Summary of Guidelines for the Use of Pre-Exposure Prophylaxis for HIV in Korea. *Infect Chemother* **49**, 243-246 (2017).

42. Organization for Economic Co-operation and Development (OECD) Health expenditure and financing - Korea <http://stats.oecd.org/Index.aspx?DataSetCode=SHA> (2015).

43. Health Insurance Review & Assessment Service Fee schedule <https://www.hira.or.kr/rd/insuadtcrtr/InsuAdtCrtrList.do?pgmid=HIRAA030069000400> (2016).

44. Weinstein, M. C. *et al.* Principles of good practice for decision analytic modeling in health-care evaluation: report of the ISPOR Task Force on Good Research Practices--Modeling Studies. *Value Health* **6**, 9-17 (2003).

45. KEB Hana Bank Average exchange rate <https://www.kebhana.com/cont/mall/mall15/mall1501/index.jsp?_menuNo=23100> (2017).

46. Fryback, D. G. *et al.* The Beaver Dam Health Outcomes Study: initial catalog of health-state quality factors. *Med Decis Making* **13**, 89-102 (1993).

47. Schacker, T., Collier, A. C., Hughes, J., Shea, T. & Corey, L. Clinical and epidemiologic features of primary HIV infection. *Ann Intern Med* **125**, 257-264 (1996).

48. Daar, E. S. *et al.* Diagnosis of primary HIV-1 infection. Los Angeles County Primary HIV Infection Recruitment Network. *Ann Intern Med* **134**, 25-29 (2001).

49. Bollinger, R. C. *et al.* Risk factors and clinical presentation of acute primary HIV infection in India. *Jama* **278**, 2085-2089 (1997).

50. Honiden, S. *et al.* The effect of diagnosis with HIV infection on health-related quality of Life. *Qual Life Res* **15**, 69-82 (2006).

51. Holtgrave, D. R. & Pinkerton, S. D. Updates of cost of illness and quality of life estimates for use in economic evaluations of HIV prevention programs. *J Acquir Immune Defic Syndr Hum Retrovirol* **16**, 54-62 (1997).

52. Tengs, T. O. & Lin, T. H. A meta-analysis of utility estimates for HIV/AIDS. *Med Decis Making* **22**, 475-481 (2002).
